# Cranial nerves involvement in craniosynostosis: a systematic review

**DOI:** 10.1007/s00381-026-07308-7

**Published:** 2026-05-07

**Authors:** Renzo Manara,, Anna Tietze, Roberto Faggin, Silvia Valeggia, Peter Tarnow, Patrizia Trevisi, Cosimo De Filippis, Irene M. J. Mathijssen, Davide Brotto

**Affiliations:** 1 Neurosciences Department, University of Padova, Padua, Italy; 2 DIMED, University of Padova, Padua, Italy; 3 Institute of Neuroradiology, Corporate Member of Freie Universität Berlin, Humboldt-Universität Zu Berlin, Berlin Institute of Health, Charitéplatz 1, 10117 Berlin, Germany; 4 Neurosurgery - Azienda Ospedale Università Padova, Padua, Italy; 5 Department of Plastic Surgery, Institute of Clinical Sciences, Sahlgrenska Academy, Gothenburg University, Sahlgrenska University Hospital, Gothenburg, Sweden; 6 Section of Otorhinolaryngology, Neurosciences Department, University of Padova, Padua, Italy; 7 Neurosciences Department, University of Padova, Padua, Italy; 8 Department of Plastic and Reconstructive Surgery and Hand Surgery, Erasmus MC-Sophia Children’s Hospital, University Medical Center Rotterdam, Rotterdam, the Netherlands; 9 Department of Developmental and Social Psychology, University of Padova, Padua, Italy; 10 Neuroradiology Unit, Azienda Ospedale Università Padova, Via Giustiniani 2, 35128 Padua, Italy

**Keywords:** Craniosynostosis, Optic nerve, Cranial nerves, MRI, Crouzon, Apert

## Abstract

**Background:**

The improved management of craniosynostosis has let emerge concomitant anatomical, functional and clinical features that are frequently faced later in life (especially by syndromic patients) and might variably encompass the cranial nerves due to stenosis of bone foramina, abnormal intracranial pressure, or anomalous gene-driven development. Clinical consequences vary according to the affected nerves, the severity and the pathogenesis of nerve involvement but also might depend on early appropriate treatment. Vision, smell and hearing, but also feeding, swallowing and facial mimic or esthesia might be affected with a possible dramatic impact on the overall development of the child and on its quality of life.

**Methods:**

A systematic literature review regarding cranial nerves involvement in craniosynostosis was performed, including case series and case reports. According to PRISMA criteria, PubMed and Scopus were searched up to February 2025 by two independent reviewers. Relevant English-language case reports and case series were included, while duplicate or aggregated data were excluded. Reference lists were screened, and disagreements were resolved by consensus. Sixty-three papers were considered. Data extracted from the papers were subjected to statistical analysis only for the optic nerve, owing to the paucity of data concerning the other cranial nerves.

**Results:**

Optic nerve involvement was reported in 140 patients (44 papers) and included papilledema (69 patients), optic nerve atrophy (69 patients), and optic nerve hypoplasia (5 patients). Visual function was reduced in 65 patients, normal in 9. In sixty-eight patients (49%) an underlying syndrome was specified, most commonly Crouzon syndrome (27/68, 39.7%) and Apert syndrome (23/68, 33.8%); 5/140 (4%) had a secondary craniosynostosis. When reported, there was a prevalence of male sex (43/67 males; 65.2%), bilateral optic nerve involvement (66/78 patients; 84.6%) and multisutural involvement (37/52; 71.2%).

Other upper cranial nerve involvement (III, IV, VI) was more frequently reported in the management of craniosynostosis children. Some cranial nerves were part of specific syndromes (e.g., I or V) or were involved during surgery. Finally, very scarce literature reports inferior cranial nerve involvement even though posterior cranial fossa neural impingement is a common complication in syndromic forms.

**Conclusion:**

Cranial nerve involvement is common; it presents differences among syndromes and specific suture involvement but is still under-investigated in craniosynostosis. Guidelines for the assessment and the proper treatment of cranial nerve–related deficits are warranted, and a joint effort of referral centers is needed for overcoming the rarity of craniosynostosis forms.

**Supplementary Information:**

The online version contains supplementary material available at 10.1007/s00381-026-07308-7.

## Background

Craniosynostoses are a large group of disorders sharing a common feature: the premature closure of one or more skull sutures resulting in deficient growth of the cranial vault during physiological brain development. This condition might be non-syndromic (with single or multisutural involvement, prevalence about 1/2600), or part of a syndromic spectrum (usually multisutural involvement with additional congenital anomalies, prevalence about 1/26,000 births) [1].

Early surgical treatment, with the primary goal to provide sufficient space for the developing brain structures and to prevent complications, has resulted in considerably improved overall survival, especially for the syndromic cases. The improved life expectancy, together with the systematic and ongoing collation of clinical, genetic, and imaging data allow to describe additional features of craniosynostosis that further characterize their phenotypic spectrum [2, 3]. These features are frequently diagnosed later in life (especially in syndromic patients) and have previously often been neglected, being overshadowed by major consequences of premature suture fusion. Among these anomalies, cranial nerves can be anomalous for several reasons: first of all because they have to exit the skull through bone foramina that may be stenotic or abnormal [4]; second, as a consequence of abnormal (increased or decreased) intracranial pressure before or after surgery, posterior cranial fossa brain structure impingement, or hydrocephalus; third, craniosynostosis is often a result of genetic alterations that also may influence brain development, including cranial nerves. Clinical consequences of cranial nerve involvement are variable depending on the affected nerves, the severity, and the pathogenesis of nerve involvement as well as the time of treatment. Vision, smell, and hearing, but also feeding, swallowing, and facial mimic or esthesia can be affected with a possible dramatic impact on the overall development and quality of life of the child.

Since our knowledge on this topic is mainly driven by anecdotal case reports and sparse case series and studies, we performed a thorough literature review on cranial nerve involvement in craniosynostosis.

## Materials and methods

Pubmed database and Scopus were screened up to February 2025, using the following keywords and meshes: “craniosynostosis” AND “cranial nerve”; “craniosynostosis” AND “cranial” AND “nerve”; “craniosynostoses” AND “cranial nerve”; “craniosynostoses” AND “cranial” AND “nerve”; in addition, each cranial nerve was searched in association with craniosynostosis in Pubmed and Google Scholar. Mendeley was used to remove duplicates. www.google.com was eventually screened for additional results. The literature search was performed by two authors independently (SV and RM), according to PRISMA criteria.

All the retrieved publications were evaluated to identify the most relevant ones. Case reports and case series were considered. Duplicated or aggregated data were excluded; only articles in English were included. The reference lists of selected articles were also analyzed to identify additional studies.

Any disagreements between the authors regarding article eligibility were resolved by consensus.

A total of 63 articles were included. The authors’ own clinical experience was used to critically discuss the data and for imaging illustrations, not including it in the data analysis. Data extracted from the papers were subjected to statistical analysis only for the optic nerve, owing to the paucity of data concerning the other cranial nerves. Studies describing involvement of cranial nerves other than the optic nerve are provided in the online supplementary materials.

Analysis included descriptive statistics, chi-squared test, and Fisher’s exact test. Due to the fragmentation of available data, a multivariate analysis was not feasible.

## Results

**Fig. 1:**
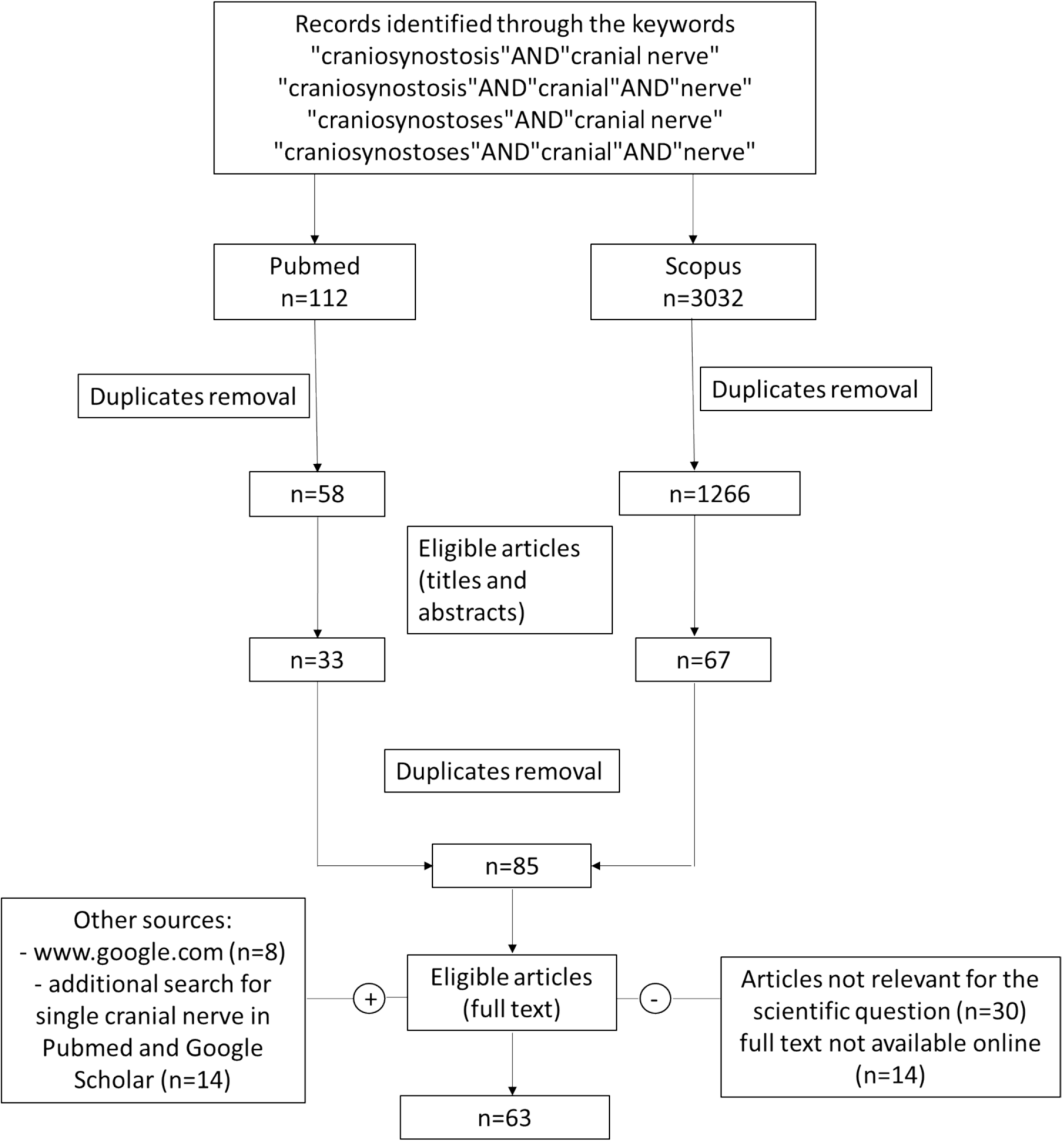
Literature search results according to PRISMA criteria

A total of 63 articles were included, of which 44 related to the optic nerve. The results of the article selection process are summarized in Fig. 1.

Due to the scarcity of literature concerning the other cranial nerves, only data related to the optic nerve underwent statistical analysis (44 papers published from 1985 up to 2024, including a total of 140 patients with optic nerve involvement in craniosynostosis). Among the latter, 68 patients (48.6%) had a confirmed syndromic diagnosis, most commonly Crouzon syndrome (27/68, 39.7%) and Apert syndrome (23/68, 33.8%), followed by Pfeiffer syndrome (9/68, 13.2%), Crouzon-like phenotypes (4/68, 5.9%), Saethre–Chotzen syndrome (3/68, 4.4%), Muenke syndrome (1/68, 1.5%) and Greig cephalopolysyndactyly syndrome (1/68, 1.5%). In five cases, craniosynostosis was secondary to systemic disorders: one case of Graves’ disease, two osteopetrosis, one rickets, and one mucopolysaccharidosis type I.

Males (*n* = 43; 65.2%) with craniosynostosis more frequently presented with optic nerve involvement than females (*n* = 24; 36.4%). The average age at diagnosis of craniosynostosis (available in 43 patients) was 5.6 ± 6.5 years. Fifteen patients had a single-suture synostosis (73.3% sagittal synostosis), while 37 patients had a multi-suture involvement.

The optic nerve involvement was bilateral in 66 patients, unilateral in 12 (four on the left side, three on the right side, unreported side in five).

The optic nerve involvement included papilledema (69 patients), optic nerve atrophy (69 patients), and optic nerve hypoplasia (5 patients).

Thirty-five percent of patients with papilledema had a history of previous surgery.

Optic nerve atrophy without increased intracranial pressure was found in 3/34 patients.

The optic nerve was assessed using various techniques, with some patients undergoing more than one assessment. The most frequently employed methods were ophthalmoscopy (57.6%) and visual evoked potentials (22.6%), followed by Optical Coherence Tomography (9.4%), ultrasound (5.7%), MRI (2.8%), and CT (1.9%).

Visual function was reduced in 65/74 (87.8%) patients and was normal in 9 (12%).

Optic nerve atrophy was significantly associated with reduced visual function (*p* = 0.02, *n* = 58), whereas papilledema was not.

Reduced visual function in patients with optic nerve involvement was common in Apert (12/12, 100% of patients), Crouzon (18/19, 94.7%), and Pfeiffer (6/7, 85.7%), whereas no cases of reduced visual function were reported in patients with Saethre–Chotzen (2 patients) or Muenke (1 patient) syndromes (*p* < 0.001; *n* = 44).

A significant difference (*p* = 0.001; *n* = 49) in the presence of optic nerve atrophy was observed among the various syndromic forms of craniosynostosis. Optic nerve atrophy was more common in Apert (100% of patients), Crouzon (83.3%), Crouzon-like (66.7%), and Pfeiffer (62.5%) syndromes, but was not found in any patient with Saethre-Chotzen or Muenke syndrome.

Data on the other cranial nerves are reported narratively in the discussion section.

## Discussion—cranial nerves

### Olfactory nerve (I)

**Fig. 2:**
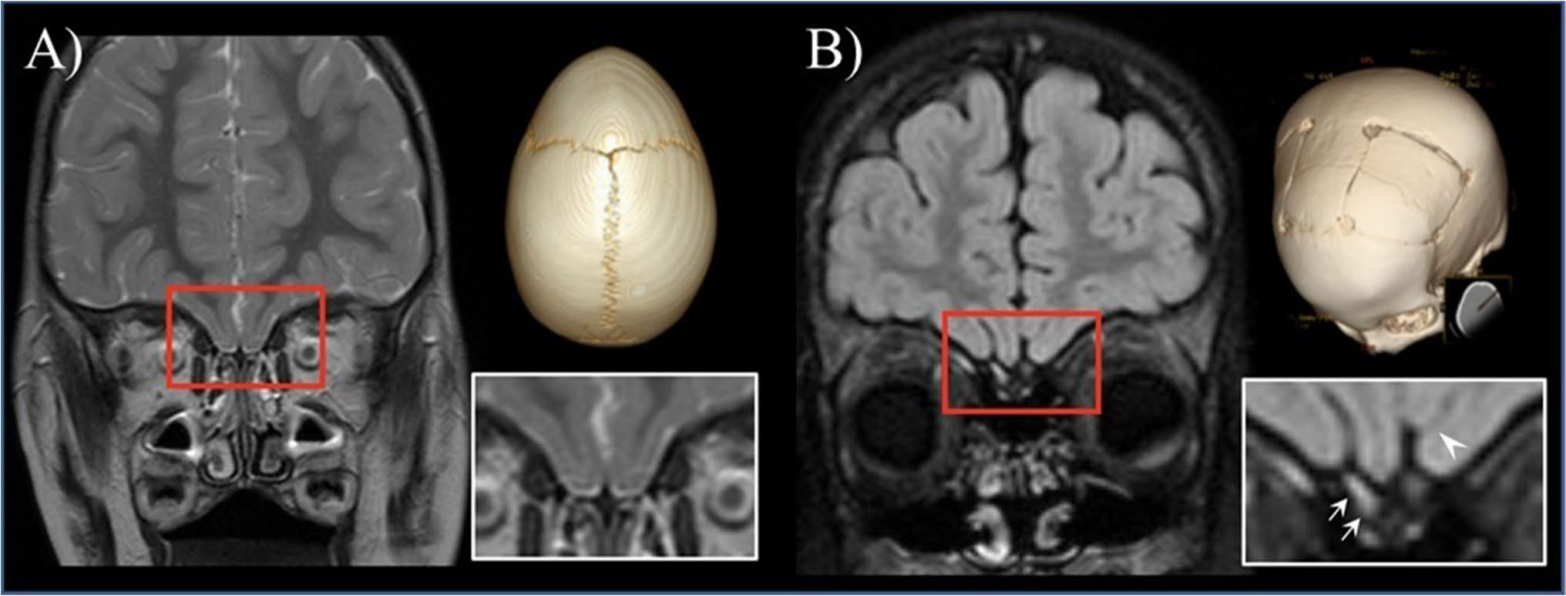
**A** Coronal T2-image in a 3-year-old boy with trigonocephaly showing bilateral agenesia of the olfactory bulbs and absence of the olfactory sulci. **B** Coronal FLAIR image in a 6-year-old boy with non-syndromic multisutural craniosynostosis showing left olfactory bulb agenesia with hypoplasia of the ipsilateral olfactory sulcus (arrowhead, note the origin from the midline and its diagonal course). The right olfactory bulb (small arrow) and olfactory sulcus are normal.

Olfactory nerve and rhinencephalon abnormalities have been reported in craniosynostosis. In 1971, Tessier stated that “anosmia is said to be frequent in patients of Crouzon disease.” [5] Hypo-anosmia may be related to anatomical abnormalities, as craniosynostosis is associated with an increased incidence of choanal atresia or pyriform aperture stenosis [6]. In addition, these patients often undergo surgical procedures involving the skull base and midface, which may traumatize the cribriform plate and thereby impair olfactory function. Moreover, an abnormally developed rhinencephalon has been described at autopsy in a newborn with Apert syndrome: the “crista galli was rudimentary and the cribriform plate was imperforate…. Olfactory bulbs and tracts were absent.” [7] Subsequent reports were published describing olfactory bulb and tract abnormalities on MRI in a child with acrocallosal syndrome [8]. Notably, the development of the anterior portion of the olfactory sulcus is induced by the olfactory bulb development. Therefore, the anatomy of the inferior frontal cortex can be used to differentiate developmental from acquired atrophy/absence of olfactory bulbs. In our experience, craniosynostosis patients may show either bilateral agenesis of the olfactory bulbs and tracts with olfactory sulcus hypoplasia, or unilateral severe hypoplasia of the olfactory bulb and tract with ipsilateral olfactory sulcus hypoplasia (Fig. 2).

However, the relationship between olfactory bulb aplasia and hypo-/anosmia remains unclear. In some conditions (e.g., tuberous sclerosis), patients with congenital olfactory bulb aplasia on MRI can have preserved smell function [9], while in other conditions (e.g,. Kallmann syndrome), normal or mildly hypoplastic olfactory bulbs are often associated with anosmia [10]. One of our craniosynostosis patients with olfactory bulb aplasia showed a normally developed cribriform plate at CT. The patient was lost to follow-up, and olfactory function could not be tested.

### Optic nerve (II)

Vision impairment in craniosynostosis was well documented almost from the very first descriptions of the skull abnormalities themselves [11, 12]. The association of exophthalmos with shallow orbits in a misshapen skull [13], blindness with scaphocephaly [14], or optic nerve atrophy with oxycephaly [15] have all been reported already in the nineteenth century revealing also the diverse pathogenesis of optic pathway involvement [16].

Optic nerve dysfunction caused by raised intracranial pressure was recognized as early as 1913, when Larsen reported that 21% of the boys at the Royal Institute for the Blind in Copenhagen had craniosynostosis.

Vision impairment is known to affect the majority of patients with multisutural defects or syndromic craniosynostosis [17, 18] based on heterogeneous, partly overlapping reasons, such as pre- or post-surgical abnormal intracranial pressure with optic nerve injury, strabismus, optic nerve hypoplasia, ametropia, amblyopia, nystagmus, or exposure keratosis [19].

In our literature review focused on optic nerve involvement, data on both suture involvement and visual function were available for only 24 patients. Among these, 23 exhibited reduced or impaired visual function, most of whom (74%) had multiple suture involvement. When considering syndromic synostosis, reduced visual function was common in Apert (100% of patients), Crouzon (94.7%) and Pfeiffer (85.7%), whereas no cases of reduced visual function were reported in patients with Saethre–Chotzen or Muenke syndrome, showing a syndrome-specific pattern of visual involvement (*p* < 0.001) (Table 1).

**Table 1:** Reduced visual function across different syndromes

Syndrome	Reduced visual function		Total
	No	Yes	
Crouzon	1	18	19
	5.3%	94.7%	100.0%
Apert	0	12	12
	0.0%	100.0%	100.0%
Saethre-Chotzen	2	0	2
	100.0%	0.0%	100.0%
Pfeiffer	1	6	7
	14.3%	85.7%	100.0%
Crouzon-like	2	1	3
	66.7%	33.3%	100.0%
Muenke	1	0	1
	100.0%	0.0%	100.0%
Total	7	37	44

Among 141 patients with syndromic craniosynostosis, Khan et al. documented in 65% of cases visual impairment of at least one eye (acuity equal to or less than 20/40) and in 40% of both eyes [20]. These data are impressive but are likely to still underestimate the true prevalence of visual impairment. In fact, in almost all craniosynostosis studies, only visual acuity has been evaluated, while measures such as contrast sensitivity, color vision, and visual field assessment have been poorly investigated. According to Liasis et al. [21], visual field defects affect up to 100% of patients with syndromic craniosynostosis. Patients are often unaware of these visual defects that go often unrecognized in visual acuity testing or visual evoked potential evaluations, while a more in-depth ophthalmologic evaluation should be performed routinely at least in syndromic craniosynostosis. In addition, visual field defects seem to present with suggestive patterns (nasal, infero-nasal and whole visual field deficits were the most consistent finding in Crouzon, Apert or Pfeiffer syndrome, respectively) suggesting the existence of syndrome-related yet unidentified factors affecting the vision function. No literature data are available on visual field assessment in single suture or multisutural non-syndromic patients leaving a large space for systematic, future research.

Notably, progressive visual deterioration in craniosynostosis patients is assumed to be of intracranial origin, thus representing a potential clinical marker of intracranial pressure changes during patient follow-up. However, pre- and postoperative visual acuity data can sometimes be misleading as they might improve before craniofacial surgery in spite of increased intracranial pressure. In fact, children tend to improve their visual acuity in behavioral tasks with increasing age. In addition, most craniosynostosis patients are very young at vision impairment onset and many do not notice the deterioration until it becomes very severe. Among patients reported in the literature, optic nerve atrophy was significantly associated with reduced visual function, whereas papilledema was not. While papilledema may represent an early manifestation of intracranial hypertension, the associated visual impairment is often less pronounced and may remain undetected in very young patients or when only limited testing is performed. Nevertheless, it carries the potential to progress to optic atrophy and irreversible visual loss if left unrecognized or untreated. For these reasons, detailed ophthalmologic evaluations should be routinely performed, both to investigate the involvement of the optic pathway and to monitor potential signs of intracranial pressure abnormalities.

Papilledema diagnosed by fundoscopy may be the sole manifestation of elevated intracranial pressure. Papilledema is detected in about 10–15% of children with non-syndromic single-suture craniosynostosis (especially sagittal synostosis) and in 30–40% of syndromic or multi-sutural non-syndromic craniosynostosis [18]. Early decompressive surgery (within the first year of age) seems to prevent the development of elevated intracranial pressure [22] and to prevent or revert the occurrence of papilledema. Increased intracranial pressure can also be observed after vault expansion [23] as 24 out of 69 patients with papilledema (34.7%) had a history of surgery. Yearly fundoscopy is recommended to detect noninvasively early signs of intracranial pressure changes. However, optic disc appearance is inconsistent and sometimes misleading, as it does not always represent a reliable indicator of increased intracranial pressure. Craniosynostosis children can show various changes of their optic disc before surgery, ranging from unilateral to bilateral or sectorial swelling or no swelling at all, but pallor. The sensitivity of papilledema is low, as it can be absent in up to two thirds of patients with elevated intracranial pressure (10/52 patients in the literature). Kim et al. observed that among those requiring a second surgery for proven increased intracranial pressure, papilledema was observed in only 50% of cases [24]. In addition, papilledema may not revert in spite of normalized post-surgical intracranial pressure illustrating its multifactorial and partly elusive pathogenesis [25]. Osseous optic nerve canal stenosis [26] or abnormal vascular perfusion may be contributing causes that do not directly benefit from the classic surgical approaches. Finally, papilledema can also be caused by intracranial pressure changes after craniosynostosis corrective surgery or be related to concomitant factors such as glycosaminoglycan deposition in mucopolysaccharidosis patients.

**Fig. 3:**
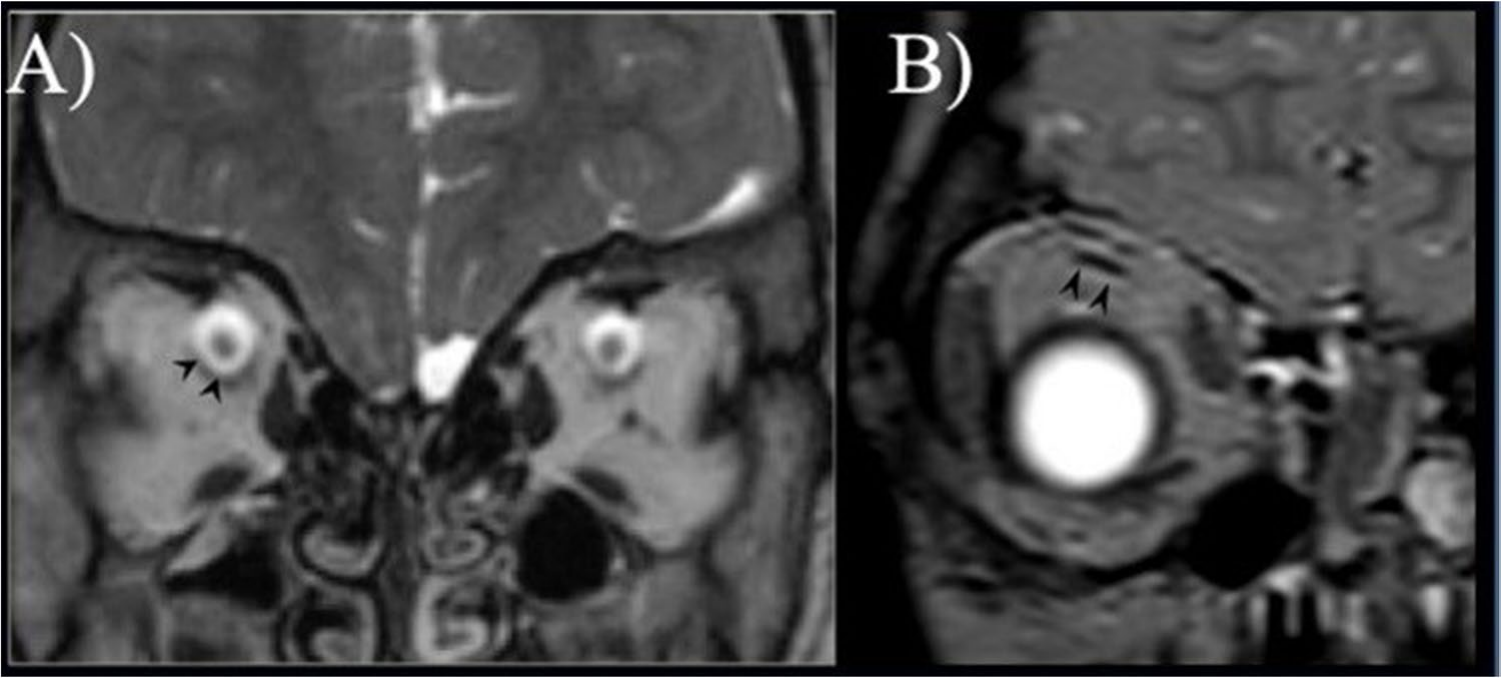
**A** Coronal T2-weighted image showing the typical target-like appearance of the optic nerve-meningeal sheet complex with the dark optic nerve in the center surrounded by hyperintense perineural cerebrospinal fluid externally lined by the thin dark dural meningeal layer (arrowheads). A diameter greater than 5–6 mm (according to age below or above 1 year) suggests enlargement of the cerebrospinal fluid space due to intracranial hypertension. On the contrary, an optic nerve diameter smaller than 3 mm suggests optic nerve atrophy or hypoplasia. **B** Coronal T2-weighted image of the right orbit showing a thin superior rectus muscle consistent with severe hypotrophy

Irreversible visual loss is one of the most feared complications of craniosynostosis-related increased intracranial pressure and its early detection has become a major focus in craniosynostosis management. As fundoscopy can be unreliable in non-compliant children, measurement of the optic nerve sheath diameter has been repeatedly proposed as a possible index of abnormal intracranial pressure. In a previous study, optic nerve sheath diameter measured 3 mm behind the globe on both CT and MRI, showed that a diameter greater than 5 or 6 mm (according to age below or above 1 year; Fig. 3A) is a valuable indicator of raised intracranial pressure [27]. However, the site of measurement or the diameter cut-off have not been standardized; in addition, the wide inter-subject variability and the need of sedation limit its widespread clinical implementation. In a series of 128 children with syndromic craniosynostosis, optic nerve sheath diameter measurements showed a high specificity of 97% for the detection of papilledema, but a low sensitivity of only 11% [28].

Recently, ultrasound evaluation and optical coherence tomography (OCT) have been proposed as valid alternative diagnostic tools. OCT showed up to 90.0% sensitivity and 81.3% specificity for detecting intracranial pressure above 20 mmHg when using maximal retinal nerve fiber layer thickness and maximal anterior projection [29]. It has recently been confirmed that OCT is superior to fundoscopy for the detection of raised intracranial pressure in syndromic and complex craniosynostosis [30]. In addition, lower macular volume on OCT seems to correlate with optic atrophy and worse visual acuity [31]. Despite growing evidence, guidelines for the widespread use of OCT are still lacking.

Chronically elevated intracranial or perineural pressure eventually leads to optic atrophy as a result of progressive neuronal death and, thus, almost non-reversible severe visual loss (so called papilledema-optic nerve atrophy sequence).

Optic nerve atrophy usually occurs in about one out of eight syndromic craniosynostosis patients at an average age of 10 years but it is unevenly distributed among syndromes; it is rare in Saethre-Chotzen or Muenke syndrome, while it is common in other syndromes such as Apert (7.8%), Pfeiffer (23.1%) or Crouzon (27.9%) [32]. This heterogeneity is confirmed by our review and partly reflects the presence of intracranial hypertension. In previous literature [33] among several clinical and imaging variables, downward cerebellar tonsil displacement (Chiari 1 malformation or cerebellar tonsil herniation due to undergrowth of the posterior cranial fossa, supratentorial hydrocephalus, intracranial venous hypertension associated to jugular vein stenosis, etc.) was the only parameter that was significantly associated to optic nerve atrophy.

Additionally, optic nerve manipulation or periprocedural ischemia during surgery might lead to neuronal damage. Indeed, severe optic nerve injury has been observed in 2/86 (2%) Le Fort osteotomies in syndromic craniosynostosis and may be associated with bleeding complications during surgery [33]. Some authors reported optic nerve atrophy without increased intracranial pressure [34] (10% in our series) or in association with anecdotal concomitant intracranial lesions (e.g., suprasellar intracranial cyst) [35]. Finally, optic nerve hypoplasia has also been described in craniosynostosis. In our series, optic nerve hypoplasia was reported in five out of 140 patients (3.6%), including one patient with Pfeiffer, one with Apert, and one with Greig cephalopolysyndactyly syndrome [36]. The association might be incidental; however, certain forms of syndromic craniosynostosis have been associated with septum pellucidum anomalies, and the optic nerve hypoplasia-septum pellucidum abnormality association is part of the holoprosencephaly spectrum (septo-optic dysplasia) [37–39]. So far, no study investigated the association between optic nerve and septum pellucidum anomalies in craniosynostosis, even though in our review, 3 of 5 patients with optic nerve hypoplasia had a confirmed diagnosis of septo-optic dysplasia (one with Apert syndrome, one with single-suture and one with multi-suture synostosis) [37–39], and a fourth patient, with Pfeiffer syndrome, presented intracranial findings suggestive of septo-optic dysplasia [40].

### Oculomotor, abducens and trochlear nerves functional involvement (III, IV, VI)

#### Congenital strabismus

Strabismus is commonly reported in craniosynostosis affecting over 60% of patients (39–76%) [19], thereby representing a major cause of amblyopia in this population. This finding is more common in syndromic craniosynostosis and in unicoronal synostosis, where the affected suture provokes a shortening of the orbital roof [41]. Strabismus is frequently divergent and when bilateral it often manifests as an “excyclotorsional syndrome” with a V-pattern exotropia that is more pronounced in upgaze [41]. The underlying mechanism is likely to be multifactorial and assumed to be mainly related to a reduced length of the unreflected part of the superior oblique muscle and the increased angle between the reflected part of its tendon and the ocular axis leading to superior oblique muscle impairment with ocular torticollis [42]. Abnormal insertion and displacement [42] or even absence of oblique and rectus muscles has also been repeatedly shown [43–46]. In murine models of Saethre-Chotzen syndrome, heterozygous loss of function of the TWIST1 gene has been shown to disrupt extraocular muscle formation and innervation [47]. For these reasons, any corrective surgery of strabismus should be planned after a thorough evaluation of the orbit including all muscle position and shape (Fig. 3B). Finally, some anecdotal MRI-proven cases of VI nerve aplasia have been reported suggesting that strabismus may also be caused by abnormal nerve development [8]. Nowadays, high-resolution T2 MR images can easily show even with 1.5 T scanners the cisternal segment of the III and VI cranial nerves, while the IV cranial nerve is reliably investigated only with 3 T MR scanners. So far, no imaging or functional data are available regarding the coexistence of nerve and muscle aplasia or an alternative muscle innervation in case of nerve aplasia.

#### Acquired ophthalmoplegia/ocular muscle dysfunction

Acquired abducens and trochlear or even oculomotor nerve palsy [48] might be caused by downward displacement of the brainstem due to increased intracranial pressure requiring prompt corrective surgery (e.g., ventricular shunting [49] or cranial vault remodeling [50]). Ophthalmoplegia can be uni- or bilateral [51], isolated or associated with papilledema/optic nerve atrophy [48, 52], and might persist after surgery even though most cases slowly improve with intracranial pressure normalization. Transient abducens nerve palsy has also been reported in 1% of post-surgery craniosynostosis patients [53], possibly due to fluctuating intracranial pressure or reflecting intracranial hypotension after cranial volume expansion, sometimes even requiring deactivation or removal of bone distractors and devices [54]. In both cases, nerve stretching injuries or nerve compression in an overcrowded posterior cranial fossa are thought to result in abducens nerve palsy. Transient abducens and trochlear nerve palsy have also been noted after surgery for strabismus correction, likely due to direct nerve injury or local complications [46].

### Trigeminal nerve (V)

The involvement of the trigeminal nerve has been reported in craniosynostosis mainly as part of a rare neurocutaneous syndrome (Gómez-López-Hernández syndrome) or as a possible consequence of corrective craniofacial surgery. The above-mentioned syndrome, also called cerebello-trigeminal-dermal dysplasia, was first characterized in 1979 and encompasses ataxia, trigeminal hypoesthesia, bilateral temporo-parietal alopecia and brachycephaly, while the MRI shows rhombencephalosynapsis with pons-vermis fusion and trigeminal nerve hypoplasia [55]. A CT study showed the absence of the foramina rotunda, suggesting the aplasia of some trigeminal (sensory) branches [56]. So far, about 60 cases have been reported, but the syndrome is likely to be underrecognized [57, 58].

Trigeminal sensory deficits after craniosynostosis corrective surgery have been investigated as distal trigeminal branches are often sacrificed during fronto-orbital advancement. Wiewrodt et al. showed, however, that even after having transected supraorbital or supratrochlear nerves, children did not complain about sensory disturbances and very few reported forehead dysesthesias [59]. Similarly, Dengler et al. found no sensory deficit after supraorbital, supratrochlear, zygomaticofacial, and zygomaticotemporal nerves’ surgical injury, likely because of spontaneous reinnervation [60].

There are furthermore anecdotal reports of trigeminal neuralgia (idiopathic [61] or due to a persistent trigeminal artery [62]) and a case of Sturge-Weber syndrome with trigonocephaly [63]. Here, trigeminal involvement is probably unrelated to craniosynostosis, but the associated conditions might complicate craniosynostosis course and management.

### Facial nerve (VII)

Acquired facial nerve involvement in craniosynostosis has been reported exclusively in association with osteopetrosis, in which stenosis of the internal auditory canal and the facial nerve canal occurs, or in cases of severe cerebellar tonsillar herniation [64, 65]. Facial nerve injury is feared during corrective surgery of craniosynostosis [66] but data on transient or permanent post-surgical deficits have not been provided so far. Similarly, data on congenital facial nerve deficit are currently not found in the literature.

### Audiovestibular nerve (VIII) and hearing involvement

Hearing loss has been repeatedly reported in children with craniosynostosis, but a reliable estimate of prevalence is challenging with currently only single case reports or small case series being available. In most cases, auditory impairment is bilateral and varies greatly in terms of severity (from slight deficit to profound hearing loss), age at onset (congenital or acquired), and type. Conductive hearing loss has been reported in Crouzon, Pfeiffer, and Apert syndromes, while sensorineural involvement has been described in Saethre-Chotzen, Muenke, and craniofrontal syndromes [19].

Congenital abnormalities can affect the external and middle ear, making permanent conductive hearing loss frequent, while a minority of patients present with inner ear or neural involvement.

Conductive hearing loss has a high prevalence in Apert syndrome [67]. The external ear can be low-set or posteriorly rotated. In addition microtia, macrotia, abnormal surface configuration of pinna, or constricted external canal can occur [68, 69]. The middle ear can be affected by temporary pathologies, such as Eustachian tube dysfunction, chronic middle ear effusion, recurrent otitis media [68, 69], but also by malformations such as calcification of ligaments, high jugular bulbs, and malformed and/or fused ossicles [70], specifically stapes footplate fixation with perilymph leakage through the footplate [67] making these patients at higher risk otogenic meningitis [71]. The stapes fixation can be associated with lateral semicircular canal involvement [72]. The inner ear might present a wide cochlear aqueduct [67], cochlear hypoplasia or dysplasia, posterior semicircular canal dehiscence, dilated vestibule or malformed lateral semicircular canals [70, 73].

In Pfeiffer syndrome, an enlarged and hypoplastic middle ear cavity has been reported, some with chronic fluid collection with or without concomitant fused or hypoplastic ossicles. Others present with non-specified inner ear abnormalities [74].

Despite mice models suggesting that inner ear anomalies should be frequent in Muenke syndrome, clinical studies are lacking, so the prevalence in humans is still unknown [75].

Finally, cholesteatoma and enlarged vestibular aqueducts can occur [76]; in our literature review, the correlation with a specific phenotype could not be asserted.

**Fig. 4:**
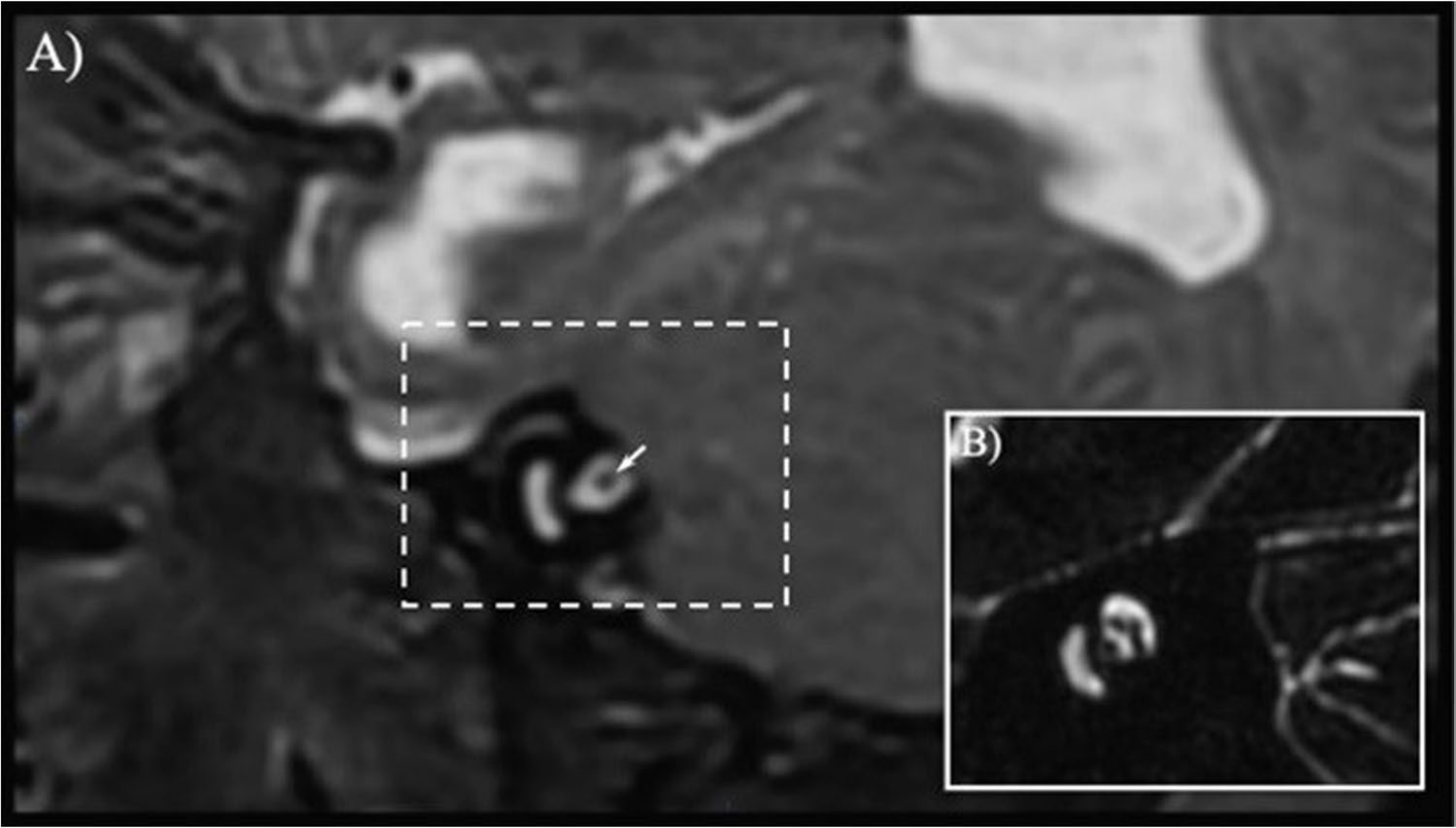
**A** Sagittal T2-weighted image in a child with Crouzon syndrome showing in the internal acoustic meatus a single dark dot (short white arrow; i.e., a single cranial nerve) presumably corresponding to the sole facial nerve due to vestibulocochlear nerve aplasia (or less likely to a fusion of seventh and eighth cranial nerves). **B** Sagittal T2-weighted image in a normal subject showing four dots in the internal acoustic meatus corresponding to the facial nerve and the cochlear, superior vestibular and inferior vestibular branches of the vestibulocochlear nerve. Partially reproduced from Valeggia et al. [73], with permission from Springer Nature (www.springernature.com)

In addition, auditory brainstem response anomalies can be detected in craniosynostosis [40], but it is still unclear whether it should be attributed to acquired or congenital central nervous system pathologies (i.e., compression or hypo/aplasia of the auditory nerve; Fig. 4). A previous report of atrophy of the VIII cranial nerve in a Crouzon patient could not differentiate between the abovementioned causes [77].

The lack of information implies the necessity to systematically characterize the auditory function and the morphology of the whole ear to be able to choose the best targeted rehabilitation measures in these patients.

### Inferior cranial nerves (IX-XII)

Impingement of the posterior cranial fossa neural structures can result in stretching or dysfunction of the inferior cranial nerves. For this reason, the onset of nystagmus, pharyngeal asymmetry, or dysphagia can indicate increased intracranial pressure requiring corrective surgery. In a recent study, inferior cranial nerve involvement was found in 26/63 (41%) craniosynostosis patients, though without significant differences among syndromes and regardless of the presence of tonsillar herniation >5 mm or syringomyelia [78]. To date, no further studies on specific involvement of inferior cranial nerves in craniosynostosis have been published.

## Limitations

The literature on the involvement of certain cranial nerves in craniosynostosis is fragmented and primarily limited to individual case reports or small case series. Therefore advanced statistics (e.g., meta-analysis) was not feasible. This study presents an exploratory review of the existing literature, offering an initial overview of the topic.

## Conclusions

Cranial nerve involvement is common, but still under-investigated in craniosynostosis. The increased life expectancy of these patients should prompt us to improve our knowledge about these aspects that may have a considerable impact on their quality of life. However, differences among syndromes or specific suture involvement prevent any generalization. Therefore, dedicated functional/clinical tests and imaging protocols should be included in the routine management of patients in order to better characterize different craniosynostosis types and provide specific guidelines for the assessment and the proper treatment of cranial nerve–related deficits. Considering the rarity of many craniosynostosis types, a joint effort across referral centers is warranted to promote our knowledge.

## Supplementary Information

Below is the link to the electronic supplementary material.

ESM 1 (DOCX.19.6 KB)

## Data Availability

No datasets were generated or analyzed during the current study.
