# Supplementary material for: Cranial nerves involvement in craniosynostosis: a systematic review
Source: Childs Nerv Syst. 2026 May 7;42(1):202. doi: 10.1007/s00381-026-07308-7 (PMC13149708; doi:10.1007/s00381-026-07308-7)
Supplement: Supplementary file 1 — (DOCX.19.6 KB) [file 381_2026_7308_MOESM1_ESM.docx]

**Online supplementary materials**

**Table 1:** cranial nerves involved in craniosynostosis (optic nerve excluded)

| **Reference** | **Cranial nerve** |
| --- | --- |
| Brocks D, Irons M, Sadeghi-Najad A, McCauley R, Wheeler P. Gomez-Lopez-Hernandez syndrome: expansion of the phenotype. Am J Med Genet. 2000 Oct 23;94(5):405-8. doi: 10.1002/1096-8628(20001023)94:5<405::aid-ajmg12>3.0.co;2-8. PMID: 11050627 | V |
| Choudhary A, Minocha P, Sitaraman S. Gomez-Lopez-Hernández syndrome: First reported case from the Indian subcontinent. Intractable Rare Dis Res. 2017 Feb;6(1):58-60. doi: 10.5582/irdr.2016.01074. PMID: 28357184; PMCID: PMC5359355 | V |
| Choudhri AF, Patel RM, Wilroy RS, Pivnick EK, Whitehead MT. Trigeminal nerve agenesis with absence of foramina rotunda in Gómez-López-Hernández syndrome. Am J Med Genet A. 2015 Jan;167A(1):238-42. doi: 10.1002/ajmg.a.36830. Epub 2014 Oct 22. PMID: 25339626 | V |
| Desai U, Rosen H, Mulliken JB, Gopen Q, Meara JG, Rogers GF. Audiologic findings in Pfeiffer syndrome. J Craniofac Surg. 2010 Sep;21(5):1411-8. doi: 10.1097/SCS.0b013e3181ebcf58. PMID: 20856029 | VIII |
| Diamond GR, Whitaker L. Ocular motility in craniofacial reconstruction. Plast Reconstr Surg. 1984 Jan;73(1):31-7. doi: 10.1097/00006534-198401000-00007. PMID: 6691073 | Innervation of extraocular muscles |
| Doerga PN, Rijken BFM, Bredero-Boelhouwer H, Joosten KFM, Neuteboom RF, Tasker RC, Dremmen MHG, Lequin MH, van Veelen MLC, Mathijssen IMJ. Neurological deficits are present in syndromic craniosynostosis patients with and without tonsillar herniation. Eur J Paediatr Neurol. 2020 Sep;28:120-125. doi: 10.1016/j.ejpn.2020.06.018. Epub 2020 Jul 21. PMID: 32782184 | Mixed |
| Gomez MR. Cerebellotrigeminal and focal dermal dysplasia: a newly recognized neurocutaneous syndrome. Brain Dev. 1979;1(4):253-6. doi: 10.1016/s0387-7604(79)80039-x. PMID: 95427. | V |
| Liu H, Katz A, Janssen P, Rajasekaran V, Stanton E, Oleru OO, Bellaire CP, Devarajan A, Napoli JG, Rutland JW, Lacoste J, Frempong T, Delman BN, Urata MM, Taub PJ. Orbital Foraminal Morphometrics in Nonsyndromic Unilateral Coronal Craniosynostosis. Ann Plast Surg. 2023 Jun 1;90(6S Suppl 5):S677-S680. doi: 10.1097/SAP.0000000000003496. Epub 2023 Mar 17. PMID: 36975106; PMCID: PMC10967031 | III, IV, VI |
| Maksem JA, Roessmann U. Apert's syndrome with central nervous system anomalies. Acta Neuropathol. 1979 Oct;48(1):59-61. doi: 10.1007/BF00691792. PMID: 506691 | I |
| Margolis S, Pachter BR, Breinin GM. Structural alterations of extraocular muscle associated with Apert's syndrome. Br J Ophthalmol. 1977 Nov;61(11):683-9. doi: 10.1136/bjo.61.11.683. PMID: 588523; PMCID: PMC1043097 | Innervation of extraocular muscles |
| Oka, Chihiro & Hirokawa, Daisuke & Homma, Hirokuni & Yasumura, Kazunori & Kobayashi, Shinji & Sato, Hironobu. (2022). Distraction osteogenesis in two cases of unilambdoid synostosis. 10.21203/rs.3.rs-1578423/v1 | VI |
| Park DH, Yoon SH. Transsutural distraction osteogenesis for 285 children with craniosynostosis: a single-institution experience. J Neurosurg Pediatr. 2016 Feb;17(2):230-239. doi: 10.3171/2015.5.PEDS14585. Epub 2015 Sep 18. PMID: 26382181 | VI |
| Pasquale Guido, Trigeminal neuralgia complicating a syndromic craniosynostosis treated with acupuncture: a case report | V |
| Perrone E, D'Almeida V, de Macena Sobreira NL, de Mello CB, de Oliveira AC, Burlin S, Soares MFF, Cernach MCSP, Alvarez Perez AB. Gomez-López-Hernández syndrome: A case report with clinical and molecular evaluation and literature review. Am J Med Genet A. 2020 Jul;182(7):1761-1766. doi: 10.1002/ajmg.a.61594. Epub 2020 Apr 17. PMID: 32302043; PMCID: PMC8988015 | V |
| Sun T, Huang Q, Li C, Wang W, He L, Liu J, Yang C. Microvascular decompression for trigeminal neuralgia caused by persistent trigeminal artery associated with craniosynostosis: a case report. J Med Case Rep. 2022 Jul 29;16(1):292. doi: 10.1186/s13256-022-03490-9. PMID: 35902917; PMCID: PMC9336082 | V |
| Tan AP, Mankad K. Apert syndrome: magnetic resonance imaging (MRI) of associated intracranial anomalies. Childs Nerv Syst. 2018 Feb;34(2):205-216. doi: 10.1007/s00381-017-3670-0. Epub 2017 Dec 2. PMID: 29198073 | I |
| Valeggia S, Dremmen MHG, Mathijssen IMJ, Gaillard L, Manara R, Ceccato R, van Hattem M, Gahrmann R. Black Bone MRI vs. CT in temporal bone assessment in craniosynostosis: a radiation-free alternative. Neuroradiology. 2025 Jan;67(1):257-267. doi: 10.1007/s00234-024-03525-6. Epub 2024 Dec 20. PMID: 39704798. | VII and VIII |
| Wiewrodt D, Wagner W. Long-term significance of injury to the supraorbital or supratrochlear nerves during frontoorbital advancement in infancy. Childs Nerv Syst. 2009 Dec;25(12):1589-91. doi: 10.1007/s00381-009-0988-2. PMID: 19763587 | V |
| Yoo H, Chung SA, Yoon SH. Abducens Nerve Palsy Following Expansion Cranioplasty with Distraction Osteogenesis. Neuroophthalmology. 2014 Oct 9;38(6):326-330. doi: 10.3109/01658107.2014.947539. PMID: 27928320; PMCID: PMC5123115 | VI |

**Table 2:** multiple cranial nerves involvement in craniosynostosis (optic nerve included)

| **Reference** | **Cranial nerve (in addition to optic nerve)** |
| --- | --- |
| Mahmoud Adel AH, Abdullah AAJ, Eissa F. Infantile osteopetrosis, craniosynostosis, and Chiari malformation type I with novel OSTEM1 mutation. J Pediatr Neurosci. 2013;8(1):34-37. doi:10.4103/1817-1745.111420. | III, VII |
| Cho DY, Evans KN, Weed MC, Lee A, Susarla SM. Bilateral Squamosal Suture Craniosynostosis Presenting with Abducens Nerve Palsy and Severe Papilledema. World Neurosurg. 2020;138:344-348. doi:10.1016/j.wneu.2020.03.079 | VI |
| Church MW, Parent-Jenkins L, Rozzelle AA, Eldis FE, Kazzi SNJ. Auditory brainstem response abnormalities and hearing loss in children with craniosynostosis. Pediatrics. 2007;119(6):e1351-1360. doi:10.1542/peds.2006-3009 | VIII |
| Dharamshi HA, Raza T, Mohsin Ali AA, Lilani Z, Ahsan SZ, Faraz A, Naqvi ST. Premature craniosynostosis in a rare genetic disease- a case report. Iran J Public Health. 2015 Mar;44(3):404-6. PMID: 25905085; PMCID: PMC4402420. | VI |
| Habibi Z, Faraji F, Mohammadi E, et al. External-internal cranial expansion to treat patients with craniocerebral disproportion due to post-shunt craniosynostosis: a case series. Childs Nerv Syst. 2023;39(4):953-961. doi:10.1007/s00381-022-05744-9 | VI (3 patients), VII |
| Kennedy D, Loh I, Branson HM, Forrest CR. Posterior Vault Distraction for Multi-Suture Craniosynostosis in a Patient with Craniometaphyseal Dysplasia: A Case Report. Craniomaxillofacial Research & Innovation. 2022;7:27528464221078443. doi:10.1177/27528464221078443 | VII |
| Kwee ML, Balemans W, Cleiren E, et al. An autosomal dominant high bone mass phenotype in association with craniosynostosis in an extended family is caused by an LRP5 missense mutation. J Bone Miner Res. 2005;20(7):1254-1260. doi:10.1359/JBMR.050303 | VIII |
| Pal US, Gupta C, Chellappa AAL. Crouzon syndrome with primary optic nerve atrophy and normal brain functions: A case report. Journal of Oral Biology and Craniofacial Research. 2012;2(2):116-118. doi:10.1016/j.jobcr.2012.03.011 | VIII |
| Paul T, Makkar SS, Mohan S, Mohan P. Metopic Craniosynostosis and Hydrocephalus in a Premature Opioid Dependent Baby. Ann Child Neurol. 2020;28(4):167-168. doi:10.26815/acn.2020.00115. | VI |
| Stavrou P, Sgouros S, Willshaw HE, Goldin JH, Hockley AD, Wake MJ. Visual failure caused by raised intracranial pressure in craniosynostosis. Childs Nerv Syst. 1997;13(2):64-67. doi:10.1007/s003810050043 | VI (2 patients) |
| Tan AP. MRI Protocol for Craniosynostosis: Replacing Ionizing Radiation–Based CT. American Journal of Roentgenology. 2019;213(6):1374-1380. doi:10.2214/AJR.19.21746 | VI |
| Diklich N, Panneerselvam S, Perez NE, Falcone M, Cavuoto KM. A novel case of Horner syndrome as the presenting sign of craniosynostosis. J AAPOS. 2024;28(2):103851. doi:10.1016/j.jaapos.2024.103851 | VI |
